# Another Letter to a Young Physician

**Published:** 1861-11

**Authors:** 


ART. III.—*Another Letter to a Young Physician; to which are appended some other Medical Papers.* By JAMES JACKSON, M.D., Prof. Emeritus of the Theory and Practice of Medicine in Harvard University. 12mo., pp. 179. Boston: Ticknor & Fields, 1861.

WE find it extremely difficult to express properly our high appreciation of this little book; a condensed record of the experience of sixty years' practice of medicine, by one universally adjudged to be entirely competent to the responsible function so long exercised; a carefully reviewed statement of the opinions and views stamped permanently on the mind of its venerated author, after all his questionings of nature and observation of varying events; the results ultimately arrived at "per tantos casus, per tot discrimina rerum."

Surely such an utterance from such a man may reasonably be considered as, in a certain sense and degree, oracular; and so, indeed, we do regard it. Dr. Jackson is everywhere known, beloved, and esteemed by our profession; and it would occupy large space if we were to repeat in set phrase the sentiments of respect and honor with which it is as well our duty as our pleasure to greet him, whenever, from the repose and retirement of his serene old age, he presents himself to claim our attention.

In the preface to this volume we have, clearly enough stated, the purpose of its publication. "Within the last year a grave question has been agitated among us, on which one whose experience has extended through more than half a century, may be thought qualified to form an opinion. The question is, whether there is any good to be derived from the practice of medicine? It is because this question has been brought before the public, that I have prepared the following Letter."

We shall proceed, with all deference, to offer a few remarks upon this Letter, from which, not only the young physician to whom it is addressed, but even the veteran disciples of the healing art, may derive profit and instruction.

It does not very clearly appear how this ancient dispute has been revived; how "the question has been again raised," or by whom it has recently been asked. Referring to the discourse delivered by Prof. O. W. Holmes, before the Massachusetts Medical Society, at their Annual Meeting, in May, 1860, Dr. Jackson observes: "Some of our brethren have regarded him as denying the utility of our art, and it was so understood by many persons not of the medical profession." But our author does not so understand the eloquent orator. He considers Dr. Holmes as merely desirous "to inculcate caution in the practice of medicine," and represents him as placing "in a strong light the evils which may be occasioned by the action of potent drugs;" and "urging us to put a reliance upon nature, and to pursue what has been called the expectant plan of treating the sick in all cases where the evidences in favor of some active practice are not clearly shown by experience." He goes on to say that he has "met the question which has in fact been entertained among us since the delivery of this discourse;" but that he has not "taken a side for or against the utility of medicine in the style of a partisan. The stand I take is somewhat that of a witness, called upon in the character of an expert." V. Preface. But in the Letter itself, page 45, he declares, "I have not forgotten that Dr. Holmes advises us to think more of the expectant treatment of diseases than we usually do. I go with him to a certain extent; perhaps as far as he does." To know precisely the position of our author, then, we must inquire what Dr. Holmes really means; *how far* he has gone; and what he actually intends by the language employed in his "Discourse."

In that document itself we have the distinct declaration, that "a body of enlightened men, organized as a distinct profession, would exist, be required, be respected, and trusted as now, whose province should be all that is now carried on by physicians, even if every drug from the vegetable, animal, and mineral kingdom were to disappear;" except, of course, that they could no longer prescribe or administer medicines. For the medical profession thus deprived, thus rendered impotent for evil, Dr. Holmes entertains and expresses a high respect. He considers that this condition of things "would leave the medical profession the most essential part of its duties, and all or more than its present share of honors; for it would be the death-blow to charlatanism, which depends for its success almost entirely on drugs, or at least on a nomenclature that suggests them."

In the discourse itself, also, we are presented with the insidious statement, in the shape of a definition, that "medicine is every unnatural or noxious agent applied for the relief of disease;" and in a note, referring to another dogma laid down in the text, that "the presumption always is that every noxious agent, including medicines proper, which hurts a well man hurts a sick one." We are advised, "that a noxious agent should never be employed in sickness, unless there is ample evidence in the particular case to overcome the general presumption against all such agents; and *the evidence is very apt to be defective.*" (The italics are ours.)

A recent essayist has very ingeniously shown that the reception of almost every proposition offered in any discussion, depends upon "how you put it;" and the remark applies well here. While speaking with mellifluous laudation of the medical profession, stripped, disarmed, and powerless, Dr. Holmes settles the whole question against their medicines, or, at least, grievously prejudices and prejudges it by "the way he puts it." If these are, as he describes and defines them, necessarily and uniformly *noxious agents*, we must beware of them, or handle them, if at all, with fear and trembling; if not in fact, virtually, with a rope round our necks. He gives them in advance a very bad character, and warns us that all evidence in their favor is "very apt to be defective." Now, we shall see that our venerable author furnishes in his Letter a good deal of such evidence, and in decided laudation of a pretty long list of these noxious agents, and is thus directly at issue with the philosopher and poet.

They have no contest surely as to the character and value of the profession, or "the utility of the art." Dr. Holmes maintains that the abolition of drugs "would leave the medical profession the most essential part of its duties, and more than all its present share of honors." On the other hand, Dr. Jackson "would hardly be willing to practice medicine without opium; would be unwilling to banish either stramonium, or even aconite, though very hazardous, (page 33;) and is fully convinced that in the early stages of diseases, powerful remedies may be employed with great benefit." Dr. Jackson, then, does not *go as far*, by any means, as Dr. Holmes.

Let us contrast the materia medica of the two physicians. That of Dr.

Holmes is comprised in a brief paragraph. It includes "Opium, with a few specifics;" which a note enumerates as follows: cinchona, mercury, arsenic, colchicum, iodine, sulphur, and antiscorbutics, and anæsthetics. In a previous note he speaks, with contemptuous irony, of specifics in general, graciously encouraging us to hope for an enlargement of the list, "when an oil is discovered that will make a bad watch keep good time; when a recipe is given which will turn an acephalous fœtus into a promising child." Wine, iron, and various salts, component elements of the body, he transfers to the materia alimentaria.

Dr. Jackson begins his pharmacopœia with the respectful mention of "Mercury, antimony, arsenic, and some other potent metallic medicines," which he "should be sorry to be deprived of; should wish to keep on the list of my materia medica." Page 16. He afterward adds ipecacuanha, squill, opium, conium, hyoscyamus, stramonium, aconite, cinchona, sulphuric ether, chloroform, iron, bismuth, cascarilla, quassia, cod-liver oil, cathartics, a large class heavily frowned upon by Dr. Holmes, colchicum, guaiacum, and "many more medicinal drugs, too good to be expunged; among the most powerful of these are elaterium, digitalis, hydrocyanic acid, and iodine; among milder drugs, spts. eth. nit., acet. ammoniæ, valerian, and assafœtida, old friends whom I would not like to part with; as also aq. ammoniæ, croton oil, and mustard, and cantharides." We have quoted this catalogue in detail, and perhaps somewhat tediously, because we think it quite as extensive a one as any modern practitioner would set down, or care to advocate; and, especially, because we were anxious to show *how far* Dr. Jackson goes beyond Dr. Holmes, in his use and estimation of "drugs—powerful drugs—noxious agents" as they are, and how much more unlikely to "throw away," cast out, and abolish the familiar apparatus medicaminum, the accumulated, perhaps profuse, material of ages.

We have a word to say, however, in passing, concerning the "presumption against medicines," as being necessarily "injurious to the sick, because hurtful to the well." It is true that many of them are disagreeable and nauseous. "Spit them out," says Dr. Holmes, following nature, as he suggests. But if nature be not always the same! To a large majority of our patients, the remedy for phthisis, at present fashionable and highly approved, cod-liver oil, is intensely disgusting. Shall "they spit it out?" The Esquimaux and the Russian esteem it a delicacy. Le Vaillant tells us that the Africans, in like manner, regard the oil from the hippopotamus as a luxury, and a valuable medicine. Every one can, from his own experience, supply similar examples of difference of taste or caprice. Nothing can be more uncertain than such a rule as this.

"A medicine, that is, a noxious agent, should always be presumed to be hurtful. It always is directly hurtful; it may sometimes be indirectly beneficial." Such is the precise phraseology of "the discourse." Is it true in any sense? Is there any meaning conveyed by it which our venerable author would assent to, or which he would be willing to be understood to sanction, when he says that he goes "with Dr. Holmes to a certain extent, perhaps as far as he does?" The whole tenor of the valuable little book before us seems rather to be exactly in opposition to, and contradictory of it, as indeed it cannot but be, conveying, as it does, the experience of a careful observer, sage thinker, and prudent practitioner. Let us examine further the terse proposition. And first, it will not be easy to find the man in full and perfect health—free as he must be, for our purpose, not only from actual disease, but from personal, hereditary, or tribal predispositions—upon whom to try the experiment of the direct hurtfulness of the noxious agent, which, when we are about to employ it for the relief of the sick, we call a medicine. When we procure such a subject we must allow for idiosyncrasy, as our first embarrassment. Some men begin the use of tobacco without a qualm, as some are never sea-sick. Next we shall discover that the influence of the deleterious substance will depend upon, or, at any rate, be prominently modified by, dose, or quantity, or concentration, or mode of presentment, or administration. No examination or analysis of it, beforehand, will aid us here; no classification, no scientific knowledge of constitution, or composition. There are but few elementary matters in nature; out of these are formed alike poisons and aliments, the difference between them being merely fractional in proportions often, and extremely tenuious. Some fungi are familiarly used as food or as condiment; others, scarcely distinguishable by the most knowing expert, and often mistaken, are terribly destructive to life; and others are exhilarating or intoxicating in a peculiar way—ascertainable only by experiment. The hemp of our Western hemisphere is inert; its congener of the East a most energetic narcotic. The chemist prepares a hydrocyanic acid of such concentrated intensity of power that the contact of an atom is fatal; as nature offers the same substance to us in many forms, we employ it familiarly to flavor our ices, cordials, cosmetics, and confectionery. From the most disgusting objects in nature we obtain the most delightful perfumes, such as we inhale gratefully from the sweetest fruits and flowers. The line is not drawn, as Dr. Holmes would have us take for granted, with any clearness, between articles possessing the most contrasted qualities; so that we can always say—here is a poison, avoid it; here a medicine, a noxious agent, directly hurtful, possibly indirectly useful, spit it out, examine the evidence concerning its ultimate influence, but remember that such evidence is extremely apt to be defective as rebutting the presumption against it: a condiment or flavoring aroma, which you may use or not, at your own taste or your pleasure : or, lastly, a food, to be sought for subsistence and nourishment. Nicotin and the oil of tobacco are most deadly, noxious agents ; but a great mass of human beings employ a large portion of it every day, in snuffing, smoking, and chewing the worshiped weed. Alcohol, taken undiluted in no very great amount, will kill a healthy man ; variously mixed in artificial formulæ it is most extensively administered to the sick as medicine ; in diverse shapes, natural and officinal, in sickness and in health, we ourselves have repeatedly experimented with it, successfully, and, thus far, safely ; and nothing in the world could give us greater pleasure than to run the risk incurred in emptying a bottle of Madeira or Champagne, or imbibing a tumbler or two of hot whisky-punch, or cool kirschenwasser in symposium with the wise, and witty, and brilliant author of the "Discourse ;" whom to read is to admire, and from whose writings we never fail to receive instruction, whether we agree with him or no. It is a sad thought that everywhere men seek out narcotics to soothe and stupefy, console and comfort, and often imbrute and degrade us. We thus forget or defy our cares, and, for awhile, distract our minds from the sorrow and suffering which oppress us. But, in the mean while, what has become of the essentially and directly hurtful character of these noxious agents ; poisons, medicines, beverages, aliments, cordials ; all of these diverse effects so characterized depending on contingencies of quantity, concentration, repetition, and adaptation ? There has been lately accumulated no small amount of evidence to show that persons in the best health may take with impunity considerable doses of arsenic—a poison, a heroic medicine, a noxious agent, often fatal in minute quantity ; often energetically active in arresting the course of disease. But we might multiply indefinitely such examples.

Another class of medicines may be pointed out which are not directly hurtful in any known mode except those in which aliments are also, (as by excessive and oppressive quantity,) which still exert a powerful influence in sickness. An infusion or tincture of cinchona will make a pleasant and innocent bitter, as harmless as tea or coffee, as a pipe or cigar ; a grain or two, or even five grains, of the concentrated principle resident in bark—quina—will not hurt or disturb a man in sound health. Yet the dose just named will cure, jugulate abruptly, or, if properly timed, prevent an attack of malarial intermittent fever by virtue of some obscure and incomprehensible property inherent in it. The same is less strikingly true of many articles, as salicine, cornine, piperine, diluted nitric acid, and a long catalogue besides, of which we cannot speak as poisons or noxious agents with any appropriate application of language. Here also we would refer to the list of cathartics. They meet with no favorable notice in the "Discourse ;" nay, we find them included in the general denunciation of medicines as noxious agents, and "directly hurtful." They are indeed unnecessary to perfectly healthy persons, who, in civilized society, are not perhaps a very large majority. But we are too well aware that a habit of constipation is among the most familiar, and, indeed, almost unavoidable results of certain conditions of civilized life. This would become serious disease in many without the relief to be obtained from them. It is an abuse of terms to say that they are only indirectly beneficial here, or that they are in any sense directly hurtful. Laxative fruits, and beverages, and aliments are innocently used every day. Excess is always and in every regard injurious; and neither we nor Dr. Jackson, who has dwelt with force and clearness on the infinite and widespread benefits to be derived from the proper exhibition of this class of remedies, would defend or excuse it. Not to speak of their value in acute maladies, we know many a student, and man of business and letters sedentary under cogency of circumstances, to whom life would be absolutely unendurable but for the orange sucked in the morning, the tablespoonful of bran swallowed at bedtime, the few grains of rhubarb, or the tumbler of Saratoga water which gave an occasional and wholesome fillip to a torpid or forgetful intestine. To depend upon nature here would be to lean upon an already broken reed, and to wait would be to wait in vain; "rusticus expectat dum defluit amnis." Thus again we find Dr. Jackson's Letter strongly in opposition to the views expressed in the "Discourse" of Dr. Holmes, notwithstanding the vague declaration of general assent volunteered in the preface.

We appreciate the little book before us, because in it the long experience of its author is made available to us and those who come after us. But if experience be in itself untrustworthy, or its deductions are without proper and logical foundation, we must be exceedingly cautious not to yield our minds to his doctrines and statements. In the "Discourse" we are ingeniously taught the lesson of the ancients, "experientia fallax;" to be sure, they also told us "experientia docet," but the emphasis must be laid on the former dogma. "Medical men are apt to suppose, after twenty years' practice," that they guide themselves and perhaps benefit others by what they have learned from observation, and regard "as the result of experience;" but "much of it"—and no one can tell how much—"is no such result at all, but a foregone conclusion, based on some prevalent belief or fashion of the time." The institution of an art built upon such shifting sands must surely crumble into ruin. Nothing is fixed or steadfast. Nay, more. Let us suppose the observations fairly made and correctly reported. The testimony of Dr. Jackson is recorded in favor of certain medicines applied as remedial in certain definite diseased conditions. He infers that the desirable changes which took place in the latter, after the administration of the former, were the effects of his treatment, consequences following causes. But Dr. Holmes denounces this course of reasoning as "inveterate logical error;" "the post hoc ergo propter hoc error; he got well after taking my medicine: therefore in consequence of taking it." Forbes urges the same repudiation of the argument, and seriously proposes "to dissociate in the minds of practitioners the notions of post hoc and propter hoc." In place, however, of this habitual, familiar, and, as it would seem, indispensable mode of reasoning, they offer us no other. They suggest no substitute, nor do they tell us how we shall guide ourselves when we abandon it. We cannot say ante hoc or sine hoc—there must be sequence, undoubtedly. If a cause does not precede, an effect cannot follow. Hume maintains that this is all that we can know of the relation. Bartlett intimates that it is useless to seek beyond it. Some have imagined that we cannot be satisfied without the conception in the mind of relevancy between them; but have not determined whether this conception is instinctive or acquired; intuitive and perfect, or educational and conjectural.

To our utter astonishment, Dr. Jackson himself long ago went astray—*pace tanti viri*—on this matter. In a letter to Sir John Forbes, published in 1846 and reproduced here, page 113, he says: "I have often urged my brethren that we should never get the better of quackery, so long as we attributed the recovery of patients to medicine on the propter hoc principle—that is, propter hoc because post hoc. Our proper ground is that having studied the subject, and had personal experience, we know better than others how to direct the cure of the sick." He would propose to introduce what is denoted, in the language of Sir John, "a more philosophical and accurate view of the relations of remedies to the animal economy and to diseases." But these relations are infinitely recondite and obscure. Does Forbes or Jackson know—is it known to any one—what is the relation of cinchona either to the animal economy or to malarious disease which qualifies it to arrest and cure intermittent fever?

Post hoc, ergo, propter hoc: "there is no way but this." We must take care not to apply our method carelessly, hastily, vaguely; but when any one event is known or observed semper et ubique et ab omnibus to follow another, we have the best ground accessible to the human intellect for the belief that the second is consequence or effect of the first as cause. We would not affirm that the method is infallible, but we say that it is indispensable and inevitable. Every inference in both the "Letters"—the import of every case recited or alluded to—are predicated upon this denounced doctrine. How else are we to understand the interesting history of Dr. Jackson's first patient, Mr. Lowell, who became very ill with "cholera infantum following (post hoc) and brought about from (propter hoc) dentition, dog-day weather, and unpropitious diet;" and restored to health after and by (post and propter) Dr. Jackson's very judicious treatment with certain noxious substances (medicines) "small doses of calomel, castor-oil, or some like article," with baths and well-selected nourishment? How else did our venerable author come to be "entirely convinced that bleeding diminishes the risk of life in young subjects laboring under pneumonitis," or that "in the adult it will somewhat shorten the disease, and more certainly diminish its violence?" How else did he arrive at the "belief that typhoid fever may sometimes be arrested at once within the first three days?" How else did his old master, Dr. Holyoke, or he himself know "that women bear bleeding better in pregnancy, much better than men, or women under other circumstances?"

Dr. Holmes humorously eulogizes the "silver hoe" which "saved the colony at Plymouth." In the case of Massasoit—"typhoid fever clearly—Winslow *scraped* his tongue with this implement," judiciously and successfully. Under a like attack, Dr. Jackson took an antimonial emetic. Page 23. He got better after it, (post hoc.) Thinking—illogically according to Forbes and Holmes—that it was because of it, (proper hoc,) he "prescribed for others what he employed for himself, and came to view it as "creating almost a certainty of a speedy and safe termination of the disease, administered on the first day." But if he had not administered it repeatedly, his inference would have been worth little or nothing. We have but one cure ascribed to the "silver hoe."

Dr. Holmes asks earnestly in the "Discourse"—" Do not some of you remember that I have had to fight this private-pestilence question"—the contagiousness of puerperal fever—"against a skepticism which sneered in the face of a mass of evidence, such as the calm statisticians of the insurance office could not listen to without horror and indignation?" Aye, indeed! We do remember it well; and now recall our supreme wonder that it did not carry conviction and remorse to the minds and hearts of those who, "while discussing the treatment of the disease, were carrying the infection from bed to bed, as rat-killers carry their poisons from one household to another." It was a most admirable treatise, of clear illustration and unanswerable argument, which was, as he says, "sneered at." Its foundation was on the strong and simple ground—post hoc, propter hoc. No relations were or could be, or need to be, pointed out "more philosophical and accurate" than the unimpugnable facts of repeated sequence; the unhappy conductor, himself clean, neat, and perfectly well, traced from a sick bed to a healthy one, which soon in its turn became a bed of pestilence; and so on, again, and again, and again, until no impartial mind could fail to recognize the connection of cause and terrible effect.

This is no digression, as it may at first seem to our readers. A general indorsement of the views promulgated by Prof. Holmes is given by the revered author of the Letter before us, as quoted from its preface. Looking upon these views as really injurious to our profession, it was incumbent upon us to deprive them of the weight of authority thus lent them by partial friendship or misapprehension. We have shown, we think, that the support was apparent rather than real. In summing up "the conclusions to which we have arrived," Dr. Jackson lays down a rule which we readily accept, and which will plainly exhibit *how far he does not go* with Dr. H. "Fifthly. When diseases are brought under the treatment of a physician, after the period during which active measures are found useful, or *when such treatment has done all that it is capable of*, the expectant mode of cure should be relied on." Page 90. Then, and not till then, we add, let the physician rely on it.

A single quotation will serve to show how widely our author differs, on a most important principle of medical practice, from Sir John Forbes, whom also he eulogizes, and seems 'promiscuously' to agree with and to sustain. Forbes urges upon 'Young Physic' the injunction, "To endeavor to banish, from the treatment of acute and dangerous diseases at least, the ancient axiom, 'Melius anceps remedium quam nullum.'" This piece of advice we have always regarded as craven and truculent. What! refuse to help a poor wretch crying out in the agonies of tetanus, or convulsed with epilepsy, or in the horrible spasms of hydrophobia, because "we are not certain of an indication!" The more acute the suffering, the more dangerous the attack, the more imperatively are we called on to exert all our energies in behalf of frail humanity. Where else is our hope of progress? Slow enough, alas! with efforts pressed enthusiastically in every direction; without such efforts there could be none whatever, and we should sit down "and wring our hands in comfortless despair."

In the true spirit of the physician, Dr. Jackson forcibly declares, "I will say that, for a disease so distressing to the patient and his friends," he is speaking of epilepsy, "a drug could scarcely be named which I would not administer, if it could be done with a reasonable *chance* of success." He would not wait for "a certain indication;" would not be repelled by an ever-present doubt.

We gladly record our author's views on another topic of pressing and paramount interest. Sir Gilbert Blane, among the numerous heresies of his mis-named "Medical Logic," asserts that "the more we study, practice, and understand our science, the longer we shall find or make the list of *self-limiting diseases.*" It is not easy to circumscribe clearly the meaning of this vague phrase. Even in Dr. Bigelow's pure English writing it is employed so as to include not only doubtful examples, but instances of the most chronic and unlimited and irregular duration. At present, the only truly self-limiting diseases we know, are the exanthemata. These *will* come to an end when they have run their brief course; we can neither protract nor renew any part of the morbid conditions which they manifest. Cancer and tuberculosis have no spontaneous limitation: it is hardly in our power to limit them. A fit of gout has a tendency to last about, or nearly, the same time at each period of its local recurrence; but the constitutional affection is illimitable: a convulsion terminates spontaneously in a few minutes, but neither epilepsy nor hysteria are constitutionally self-limiting. Intermittent fever has a well-defined paroxysm for every type, but, if let alone, will recur during a whole lifetime. We might make an enormous catalogue of doubtful maladies, of which it is affirmed and denied that they persist in a given course and cannot safely and efficiently be interfered with. From this long list which the expectant medicine would leave unabridged, or rather which it seems to seek to extend, every faithful practitioner delights to subtract. Typhoid fever, one of its prominent features, is confidently believed by Dr. Jackson to be liable to jugulation, arrest. We are happy to notice that our distinguished clinical Professor Flint has recently expressed a similar opinion. Dr. Jackson is also "inclined to say the same thing in regard to pleurisy, pneumony, and the similar affections; that they may be diminished in violence and *duration*, sometimes perhaps may be arrested, jugulated, by blood-letting, aided by appropriate remedies." Page 50. Of gout, he writes, page 72, "It has been called the opprobrium medici, and that the doctor could not remove it. Now, if this be said as to a fit of the gout, it is no longer true." And among his 'conclusions,' we find him expressing his 'conviction'—"Thirdly, that by medical treatment—often by the efficient use of powerful drugs, sometimes by blood-letting—we may frequently succeed in diminishing the violence, lessening the suffering, and shortening the duration of diseases." And once more, page 89—"Fourthly, I have brought into view the self-limiting diseases of which Dr. Bigelow has treated, in which certain processes must be gone through, and which medicine cannot arrest. I have stated that we can sometimes diminish the suffering, and perhaps also the danger, attending these diseases, when they are passing certain boundaries." We are glad to see here avowed a willingness to interfere even with the unquestioned members of the class alluded to, while we show above our author's sagacity in making subtractions from their alleged number.

We have had occasion incidentally to notice his position in regard to the disuse of blood-letting, and the recently exaggerated repugnance to antiphlogistic measures generally in our therapeutics. "I feel persuaded from my own observation and experience," he says, page 55, "that the use of the lancet should not be altogether abandoned. Even in the epidemic called peripneumonia notha, typhoid pneumonia, bilious pneumonia, etc., a vigorous man might be bled within the first few hours of the disease, not only with safety but with advantage." "When I began practice," sixty years ago and more! "I was not disposed to employ bleeding in any cases, especially in those of children. But finding that in them acute inflammations within the thorax were often fatal, I felt myself bound to make a trial of this remedy, which had been approved by many men of sound minds. I then tried bleeding in subjects of all ages, and the opinions above given resulted from these trials." Page 58. As a sound decision upon a practical point, formerly discussed much more than at present, we quote from page 88, "repeated small bleedings I regard as very injurious." He lays little stress on leeches and cupping, and, indeed, says little of them.

He does not add much to what we find in his former Letter on cathartics, but the advice he gives is prudent and valuable. "There is no tribe or class of medicines so much used as this. It would seem that we can hardly live without them, they are necessary; but the abuses in their use are enormous." Page 41. The truth is here fairly stated, and in the latter part of the sentence is shown how far he goes with Dr. Holmes. But the substitutes which the latter hints at, can never take the place of cathartic medicines. They are also evils: noxious, liable to great abuse, capable of inflicting much injury, and incapable, as a general rule, of compensating for the risk by any marked equivalent beneficial influences.

We have now concluded our inadequate notice of Dr. Jackson's Letter on 'the Utility of Medicine.' The appended 'notes' contain matter of much interest, most of which, however, has probably been for some time in the hands of our readers. No American can peruse without deep feeling the 'Memoir on the Sickness and Death of Gen. Washington,' or the article concerning Mr. Prescott; upon which we forbear to offer any comment. We have made some allusions above to the case of Mr. Lowell, the subject of the first 'note;' and will merely refer to the third as a simple and kind eulogy of the excellent Rebecca Taylor, who well deserves the fame she is doubtless surprised to have obtained from the pens of Drs. Jackson and Holmes.

Nothing, then, remains but to congratulate our readers on the addition to their libraries of this estimable little volume, and to offer our sincere thanks and most cordial regards to its honored and venerable author. May he yet live long to enjoy the respect and affection of all who know and of very many who never saw him.

